# Unraveling the functional landscape of ATRA- and DMSO-differentiated HL-60 cells

**DOI:** 10.1371/journal.pone.0331783

**Published:** 2025-09-08

**Authors:** Susanna Ghonyan, David Poghosyan, Anush Martirosyan, Sona Margaryan, Aida Avetisyan, Zaruhi Khachatryan, Gayane Manukyan

**Affiliations:** 1 Laboratory of Molecular and Cellular Immunology, Institute of Molecular Biology, National Academy of Sciences, Yerevan, Armenia; 2 Laboratory of Cell Biology and Virology, Institute of Molecular Biology, National Academy of Sciences, Yerevan, Armenia; 3 Laboratory of Evolutionary Genomics, Institute of Molecular Biology, National Academy of Sciences, Yerevan, Armenia; UNC CH: The University of North Carolina at Chapel Hill, UNITED STATES OF AMERICA

## Abstract

The short lifespan of polymorphonuclear neutrophils (PMNs) *in vitro* poses challenges, as their limited viability restricts functional assays and experimental manipulations. The HL-60 cell line serves as a valuable model for neutrophil-like differentiation, yet the functional relevance of ATRA- and DMSO-induced differentiation remains incompletely understood. In the present study, we aimed to characterize the differentiation potential of all-trans retinoic acid (ATRA) and dimethyl sulfoxide (DMSO) on HL-60 cells and compare their functionality with primary PMNs. Besides that, we performed profound immunophenotypes of the cells with multicolor cytometry, and evaluated their antitumor capabilities. Our findings indicate that both differentiation conditions yield cells resembling immature neutrophils, exhibiting promyelocyte-like morphology, lacking key maturity markers. However, ATRA-differentiated cells exhibit a more mature phenotype, with higher expression of *C/EBPα* and reduced proliferation rates, indicating advanced differentiation. Functionally, ATRA-dHL-60 cells displayed limited immune responses, showing minimal phagocytic activity, low ROS production, and a reduced response to LPS. In contrast, DMSO-dHL-60 cells, despite their less mature phenotype, showed enhanced NET formation, and tumor-promoting potential. Additionally, DMSO-dHL-60 cells demonstrated superior adhesion and migration abilities, likely due to increased expression of CD18 and CD31. Overall, different differentiation conditions shape the functional specialization of HL-60 cells, with ATRA promoting a more neutrophil-like maturation and moderate activation, while DMSO results in a more immature phenotype with enhanced NET formation. These distinct properties suggest that ATRA-dHL-60 cells may better model neutrophils in chronic inflammation, whereas DMSO-dHL-60 cells could be more suitable for studying NETosis-driven autoimmune, thrombotic disorders and cancer.

## Introduction

Neutrophils or polymorphonuclear neutrophils (PMNs) play a crucial role in the immune defense against invading pathogens through phagocytosis, intracellular degradation, release of granules, reactive oxidative species (ROS) generation and formation of neutrophil extracellular traps (NETs) [1,2]. Besides their antimicrobial and cytotoxic mechanisms, neutrophils play an important role in many aspects of tissue homeostasis, including regulation of extravasation, granulopoiesis, clearance of apoptotic or senescent neutrophils, modulation of core metabolism, etc. [3–5]. PMNs are also able to mediate innate immune responses contributing to the adaptive immune response through the promotion of T-cell activation and dendritic cell (DC) maturation by multiple pathways [6,7]. A growing body of evidence has revealed an unexpected phenotypic heterogeneity and functional versatility within the neutrophil population. Recently identified prolonged lifespan of PMNs in tissues, their capability for *de novo* cytokine synthesis, and ability to recirculate through various tissues expand their repertoire of immunomodulatory functions [8,9]. Emerging data concerning the immunomodulatory functions of PMNs underscore the necessity for conducting more thorough and comprehensive studies on their role. A robust and reliable model system is crucial for such studies, particularly one that closely mimics the functional and phenotypic characteristics of peripheral blood neutrophils.

Cell lines serve as a cornerstone for researchers, providing a standardized platform to study cellular behaviors, conduct experiments, and advance scientific understanding across diverse fields. Among them, the HL-60 cell line, characterized by its myeloblastic or promyelocytic properties, stands out as the predominant choice in PMN research [10]. HL-60 is a suspension cell line composed of cells that vary in size, occasionally exhibiting pseudopods and round nuclei [11,12]. HL-60 cells undergo differentiation into myeloid cell types, including granulocytes and monocytes. Treating HL-60 cells with agents like all-trans retinoic acid (ATRA) or dimethyl sulfoxide (DMSO) can induce their differentiation into cells with characteristics resembling neutrophils [13]. A small proportion of HL-60 cells are able to spontaneously differentiate into granulocyte-like cells which was suggested to be due to lack of the *c-Myc* gene [14]. While HL-60 cells offer an alternative to primary human PMNs, their lack of specific granules, a hallmark of PMNs, presents a notable limitation that could complicate comparisons across studies. The suitability of the HL-60 cell line differentiated by ATRA or DMSO as a model for neutrophils was initially questioned by researchers due to concerns about its ability to fully replicate the functional and phenotypic characteristics of primary neutrophils. While ATRA and DMSO are both widely used to induce HL-60 differentiation, the biological justifications for selecting one over the other are often unclear. Moreover, the literature presents conflicting reports regarding the phenotypic and functional characteristics of ATRA- and DMSO-differentiated (dHL-60) cells. These inconsistencies further complicate the choice of the most appropriate differentiation method for specific experimental assays.

This study aims to systematically investigate the differentiation potential of ATRA and DMSO in HL-60 cells, focusing on their phenotypic, morphological, and functional characteristics. We seek to comprehensively analyze the immunophenotype, NET formation, and activation status of differentiated cells, along with their responses to LPS stimulation. Additionally, we aim to assess their tumor-associated properties, including migratory behavior, interaction with cancer cells, and potential influence on tumor growth. These findings will provide insights into the suitability of ATRA- and DMSO-dHL-60 cells as neutrophil-like models for immunological and tumor-related research.

## Materials and methods

### Neutrophil isolation

Peripheral blood was collected into EDTA-containing tubes and processed within an hour after collection. PMNs were isolated by density gradient centrifugation using Histopaque-1077 (Sigma-Aldrich), followed by erythrocyte lysis. The purity of isolated cells was assessed by flow cytometry and proved to be > 95%.

The study enrolled healthy volunteers (mean age 35 ± 7.8 years) with no history of acute or chronic diseases from September 2024 to January 2025. Participants who were regularly taking any type of medication were excluded. The study was approved by the Ethical Committee of the Institute of Molecular Biology NAS RA (IRB00004079, IORG0003427). Written informed consent was obtained from all participants before sampling.

### Cell culture and differentiation

The HL-60 cell line (derived from a patient with acute promyelocytic leukemia) was purchased from ATCC. The HL-60 cells and PMNs were maintained in RPMI-1640 supplemented with 15% or 10% FBS respectively, 2 mM L-glutamine, 100 µg/mL streptomycin, 100 U/mL of penicillin (Sigma-Aldrich) at 37°C in a humidified atmosphere with 5% CO_2_. Fresh media was replenished every third day, and cells were passaged upon reaching a maximum density of 1 × 10^6^ cells/mL. For differentiation into neutrophil-like cells (dHL-60), HL-60 cells were stimulated with DMSO (1.25%) or ATRA (1 μM) during 3, 4, 5, and 6 days. The differentiation was controlled with flow cytometry and microscopy. Monocyte-like differentiation was excluded by confirming the dim expression for CD14. Neutrophil-like maturation was additionally confirmed by observing the segmentation of the nucleus into lobulated shape.

### Morphology

Morphological characterization of dHL-60 cells and PMNs was conducted using two complementary approaches: fluorescent staining and classical hematoxylin and eosin (H&E) staining for histological analysis. For fluorescent staining, cells were allowed to adhere to poly-L-lysine coated slides for 30 min, then fixed with 4% paraformaldehyde for 15 min, washed and permeabilized by 0.1% Triton-X100 (Sigma-Aldrich) for 20 min. Non-specific binding was blocked by incubating cells with 5% BSA. Fixed and permeabilized cells were incubated overnight at +4°C with primary mouse anti-human Arginase 1 antibodies (BioLegend), followed by staining with donkey anti-mouse Alexa Fluor 647-conjugated secondary antibodies (Abcam) and Alexa Fluor® 488 Phalloidin (Cell Signaling Technology). Prewashed slides were mounted with a glass coverslip using a Mounting Medium with DAPI (Abcam). All images were captured using a Cytation C10 microscope imaging system (Agilent BioTek). The images were taken at 40x magnification.

For histological H&E staining, cells were attached to the slides similarly as for fluorescent staining, washed and immediately stained with hematoxylin and eosin. Slides were mounted with a glass coverslip and images were taken using a Boeco BM-800 microscope (Germany) at 1250x magnification.

### Flow cytometry

To access the expression levels of surface and intracellular markers of primary PMNs, not-differentiated and differentiated HL-60 cells, a wide panel of antibodies was used. Full list of antibodies presented in S1 Table in S1 File. For surface staining, cells were prewashed, resuspended in PBS and incubated with an antibody cocktail for 20 min in the dark. For intracellular staining, the cells were stained for surface markers then fixed, washed with PBS, permeabilized (BioLegend), resuspended in a PBS and incubated with an antibody cocktail for 20 min. Isotype controls and fluorescence minus one (FMO) controls were employed to ascertain the positivity and negativity of marker expression. The acquisition was performed on an LSRII flow cytometer (BD Biosciences) equipped with the BD FACSDiva v.8.0.1 software. Obtained data were analyzed using FlowJo V10 software (FlowJo, Ashland, OR, USA). The results are presented as the mean fluorescence intensity (MFI) or the percentage of positive events for each examined marker.

### Viability/Apoptosis

PMNs, non-differentiated HL-60, ATRA or DMSO differentiated HL-60, were incubated with a 100 μl staining buffer containing 2 μl Annexin V-FITC (BioLegend). Afterward, cells were labeled with PI (BioLegend) and analysed within 10 min using BD FACSCalibur (BD Biosciences) equipped with the CellQuest v.5.2 software.

### Ki-67 (Proliferation of dHL-60)

HL-60 cells were differentiated for 5 days with ATRA or DMSO as described above. Differentiated cells were washed, fixed/permeabilized, stained with anti-Ki67 antibodies conjugated with Alexa Fluor 488 for 20 min and analyzed on LSRII flow cytometer. As a control, non-differentiated HL-60 cells were used.

### Total RNA extraction

Total RNA was isolated from the total of 5x10^5^ cells. For this cells were resuspended in 500 μl of TRIzol Reagent and incubated for 5 min. Afterwards, chloroform (Sigma-Aldrich) was added for 3 min, and samples were centrifuged at 12,000 g at 4°C for 15 min. The upper aqueous phase containing the RNA was transferred to a new tube. For RNA precipitation, isopropanol (Sigma-Aldrich) was added to the samples and incubated for 10 min, followed by a 12,000 g centrifugation at 4°C for 10 min. The RNA pellet was solubilised with ice-cold 75% ethanol. After centrifugation at 7500 g, supernatant was discarded and samples were air dried for 10 min before being diluted in 20 μL of RNase-free water (Qiagen). Concentration and purity of RNA was detected via Nanodrop. Samples with OD260/280 at 1.8–2.0 were used for RNA quantification.

### Reverse transcription quantitative real-time PCR

To assess the relative levels of genes expression, cDNA synthesis and probe-based qPCR was performed with the use of SOLIScript 1-step Probe Kit with the following cycling conditions: 50°C for 15 min for reverse transcription, 95°C for 10 min for initial denaturation, and 30 cycles of 95°C for 15 s and 60°C for 60 s using Rotor-Gene Q real-time PCR cycler (Qiagen, Germany), 200 nM concentration for primers were used. The primer sequences provided in S2 Table in S1 File.

### LPS stimulation assay

dHL-60 cells and PMNs were treated with LPS (100 ng/mL, Invivogen) and incubated at 37°C under a humidified atmosphere of 5% CO_2_ for 4 hours. Supernatants were collected and stored at −80°C for further ELISA assay. Harvested cells were resuspended in 200 μl PBS and stained for 20 min with antibodies against CD14 (FITC, BioLegend), CD11b (PerCP CY5.5, BioLegend), CD284 (PE, eBioscience) and CD66b (APC, BioLegend). Expression levels of surface marker expression were measured using an LSRII flow cytometer.

### ROS generation

To determine oxidative burst capacity of dHL-60 cells and PMNs, dihydrorhodamine 123 (DHR-123, Sigma-Aldrich) conversion into the fluorophore rhodamine was evaluated. The cells were treated with DHR-123 at a final concentration of 10 μM for 20 minutes at 37°C. Then the cells were stimulated with 100nM fMLP, or 100 ng/ml phorbol 12-myristate 13-acetate (PMA). for 30 min at 37°C. As a negative control, cells were left in the RPMI medium alone. The reaction was stopped by placing the cells on ice for 10 min and immediately analyzed using an LSRII flow cytometer.

Extracellular ROS production by the studied cells was assessed in the presence of 15% tumor cell-conditioned medium (CM), collected from cultured tumor cells. Cells were stimulated with (PMA, and ROS generation was monitored using luminol-enhanced chemiluminescence. This method allows detection of a broad spectrum of reactive oxygen species primarily from extracellular sources. Briefly, 100.000 cells were seeded into a white, clear-bottom 96-well microplate containing Hank’s Balanced Salt Solution (HBSS) and 100 μM luminol (Sigma-Aldrich), followed by the addition of 50 ng/mL PMA. Chemiluminescence was recorded kinetically at 37 °C using the Cytation 10 imaging reader (Agilent BioTek).

### Phagocytosis assay

Phagocytosis assay was performed using Fluoresbrite yellow-green (YG) microspheres (1μM, Polysciences) or pHrodo Green Zymosan BioParticles (Invitrogen). Prior to use, Fluoresbrite YG microspheres were opsonized with human IgG to facilitate recognition and uptake by phagocytic cells. PMNs and dHL-60 cells were left untreated or pre-treated with LPS (100 ng/ml) or fMLP (100 nM) for 30 min at 37°C. Cells were then incubated with with 25 μg/ml pHrodo Green Zymosan BioParticles (Invitrogen) or 1 × 10⁷ beads/mL Fluoresbrite YG microspheres for 1 hour at 37°C. Following incubation, cells were washed with ice-cold PBS to remove unbound (non-ingested) particles, placed on ice for 15 min and analyzed by LSRII flow cytometer.

### Ca^2+^ flux assay

Neutrophils were resuspended at 6 × 10^5^ cells in 0.5 ml PBS and loaded with calcium probe Fluo-4 AM (5 μM, Invitrogen) in the presence of Pluronic F-127 (0.02%, Invitrogen) for 30 min at 37⁰C. After loading, cells were washed and resuspended in Ca^2+^ and Mg^2+^ containing PBS and placed at 37°C for 30 min for Fluo-4 AM de-esterification. The measurement was conducted in two phases: first, a baseline measurement of unstimulated cells was recorded for 30 seconds. Then, Ca ionophore Ionomycin (1 µg/ml) was added, and the sample was immediately measured for an additional 5 minutes. For all samples, a second recording was appended to the initial measurement to track the dynamics of Ca² ⁺ flux, with an LSRII.

### Neutrophil transmigration assay

dHL-60 cells and PMNs were subjected to transmigration using 6.5-mm diameter Transwell Inserts with 3-μm pore size Polyester Membrane (Corning, NY, USA). The inserts (upper compartment) were placed into the wells (lower compartment) of a 24-well plate containing 600 μl of complete RPMI-1640 with or without fMLP (100 nM). The cells were suspended at a final concentration of 2 × 10^5^/100 μl complete RPMI-1640, loaded into the upper compartment and allowed to transmigrate for 2h at 37°C. Following this period, inserts with non-migrated cells were removed and migrated cells in the lower compartment were stained with 2.5 µg/ml Hoechst 33342 (BD Biosciences). Migrated cells were imaged using a microscope imaging system (Agilent BioTek Cytation C10) in the montage mode (9 locations) that covered the whole well surface. Counting was performed automatically with the use of BioTek Gen5 Software.

### dHL-60 and PMNs interplay with HeLa and MCF7 cells

CFSE-labeled or unlabeled HeLa (epithelial cell line derived from cervical cancer) and MCF7 (epithelial cell line derived from breast cancer), both from ATCC, were seeded in 48 well plates at 1.5 × 10^4^ and allowed to adhere overnight at +37°C and 5% CO_2_. These conditions yielded a ~ 60% confluence for both cell lines on day 1. Next day, ATRA- and DMSO-dHL-60 and PMNs were seeded separately on Hela and MCF7 in a 10 × 1 ratio. During the whole experiment, Hela cells with or without PMNs or dHL-60 were maintained in RPMI, while MCF7 in RPMI mixed with DMEM at 1:2 ratio. To measure viability of PMNs and dHL-60 cells, cells were collected from the wells after 24 h co-cultivation and stained with Annexin V and PI as described above. Viability and proliferative activity of MCF7 and HeLa cells were assessed in another set of experiments, following 2 or 3 day-long co-cultivation with dHL-60. Cells were stained with CD18, Calcein-AM and 7AAD, or Annexin and PI. All measurements were performed on the LSRII flow cytometer. Supernatants from the co-cultures were collected for the analysis of soluble mediators.

### dHL60 cell adhesion to HeLa monolayer

HeLa cells were grown to 80% confluence on 24-well plates using complete RPMI-1640 medium. Immediately before assay, the HeLa monolayer was depleted from RPMI and washed. dHL-60 cells and PMNs were stained with CFSE, resuspended at final concentration 1 × 10^5^/100 μl PBS and loaded on HeLa cells monolayer. Plate was placed on a horizontal shaker operating at 120 strokes/min and cells were allowed to adhere for 15 min. Unattached cells were removed with double PBS rinsing. Adherent cells were imaged using a microscope imaging system (Agilent BioTek Cytation C10) in the montage mode (9 locations). Counting was performed automatically with the use of BioTek Gen5 Software.

### NET formation assay

ATRA and DMSO dHL-60 cells and PMNs at 100.000 cells/100μl RPMI were placed on Poly-L-Lysine-coated slides and stimulated with PMA (50 ng/mL) for 4 hours at 37°C. After washing, the slides were stained with anti-human anti-citrullinated histone H3 (BioLegend) and mounted with a glass coverslip using a Mounting Medium with DAPI (Abcam). Images were captured using a Cytation C10, at 20x magnification.

### Actin polymerization

Spontaneous and fMLP-stimulated actin polymerization was measured by flow cytometry. Studied cells were left untreated or stimulated with fMLP (100 nM) for 2 min. The reaction was stopped by adding Fixation Buffer, followed by 20 min incubation. Cells were permeabilized and stained with Alexa Fluor 488 Phalloidin (Cell Signalling Technology, 1:1000), which specifically binds to filamentous actin (F-actin). F-actin content was expressed as mean fluorescence intensity.

### ELISA

The concentrations of IL-1β, IL-8 and TNF-α in culture supernatants were measured by commercial ELISA MAX Deluxe Sets (BioLegend). Matrix metalloproteinase (MMP-9) and transforming growth factor beta (TGF-β) levels in supernatants from co-cultures of MCF-7 cells and the studied cell populations were measured using a custom LEGENDplex bead-based immunoassay (BioLegend), following the manufacturer’s instructions.

### Statistical analyses

Data analysis was performed with GraphPad Prism v 9.3.1 software (GraphPad Software, USA). Significance was determined by unpaired t tests, paired t tests or one-way ANOVA with Tukey’s multiple comparisons test. Statistical parametric analyses were performed following confirmation of normal distribution of the data. All values are given as mean ±  standard error (SE), unless otherwise specified. Values of p < 0.05 were considered statistically significant.

## Results

### Indicators of HL-60 cell differentiation induced by ATRA or DMSO

**Fig 1:**
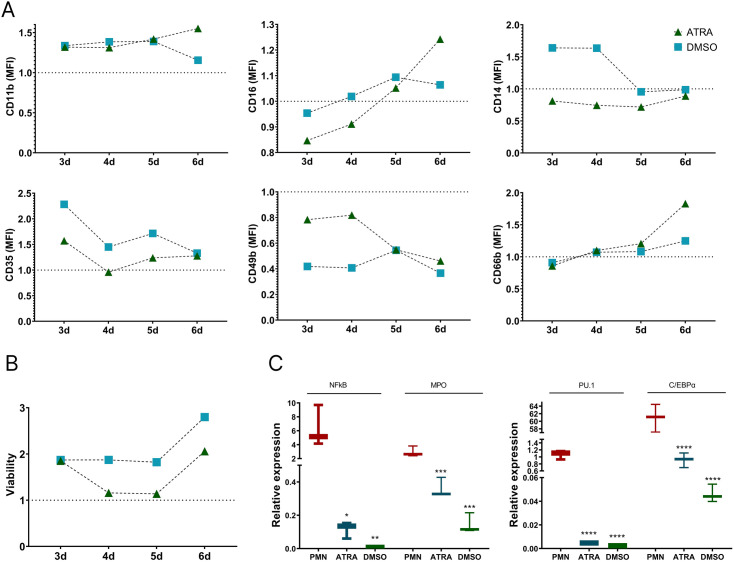
Determination of differentiation status of dHL-60 cells. A) Phenotypic analyses of HL-60 cells differentiated with ATRA and DMSO over 3-6 days. Data presented as normalisation to non-differentiated HL-60; B) Rate of cell death determined with 7AAD. Data presented as normalisation to non-differentiated HL-60; C) mRNA basal expression of *PU.1*, *C/EBPα*, *NFkB* and *MPO* in studied cells. Expression of *GAPDH* was used as reference to calculate relative expression of target genes with the 2^-ΔCt^ method. * *p < 0.05*, ** *p < 0.01*, *** *p < 0.001* – differences with the PMN group.

Initially, HL-60 cells underwent differentiation with ATRA or DMSO for 3–6 days, during which surface markers CD11b, CD14, CD16, CD35, CD38, CD66b and cell viability were assessed (Fig 1A). Analysis of cell viability revealed a significant increase in the proportion of dead cells on day 6 in both differentiation groups (Fig 1B). Given that a five-day induction period produced optimal marker expression and cell viability, dHL-60 cells were cultivated with ATRA and DMSO for five days in this study to assess their functionality. Having established an optimal differentiation protocol using ATRA and DMSO based on surface marker expression, we next aimed to analyze the transcriptional profiles of differentiated HL-60 cells with those of primary neutrophils (Fig 1C). As expected, PMNs exhibited the highest transcriptional activity among the studied cells, followed by ATRA-dHL-60 cells. Both ATRA- and DMSO-dHL-60 cells showed an absence of transcription factor *PU.1* mRNA expression, while *C/EBPα* mRNA levels were elevated in ATRA-dHL-60 cells, suggesting a more advanced stage of maturation. Additionally, DMSO-dHL-60 cells lacked *NFκB* gene expression, and exhibited the lowest *MPO* mRNA levels (Fig 1C).

### Morphological and growth characteristics of differentiated HL-60 cells

**Fig 2:**
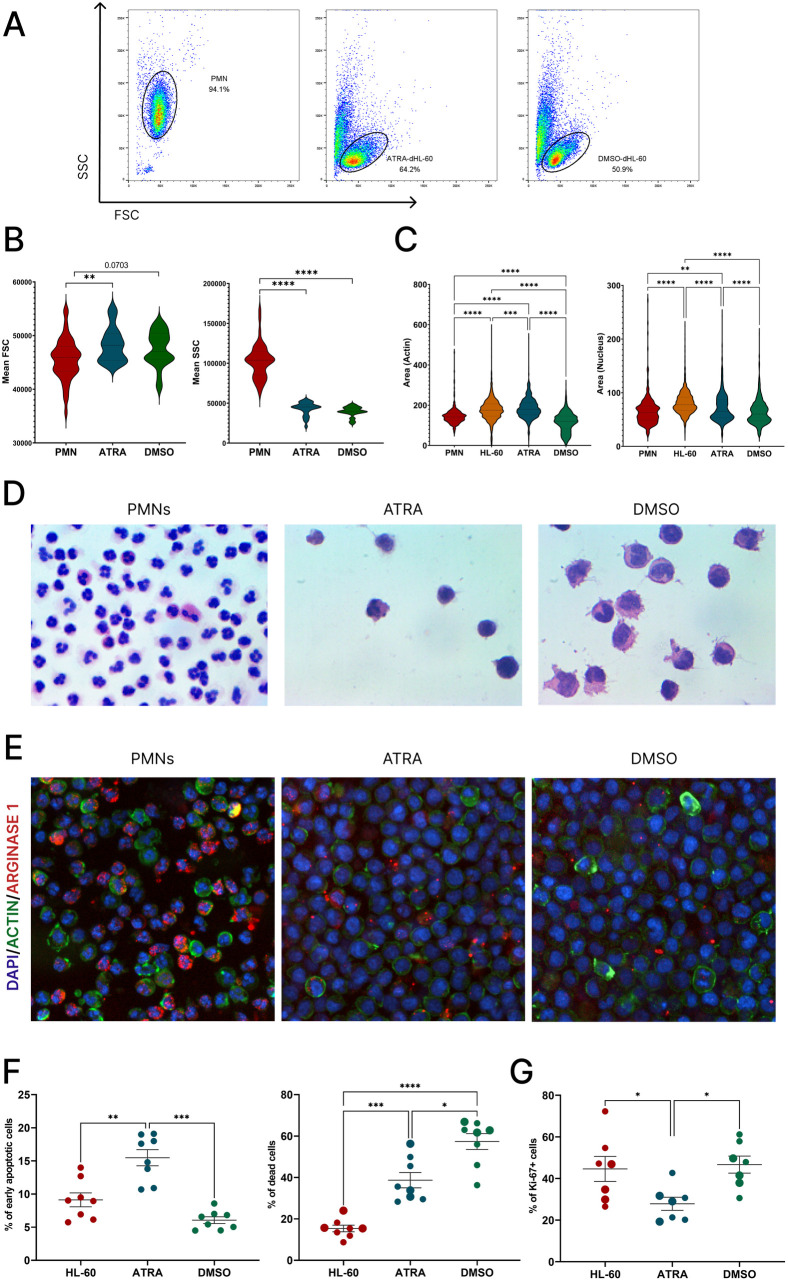
Morphology, granularity, and proliferative characteristics of PMNs, ATRA- and DMSO-dHL-60 cells. A) Gating of PMNs, ATRA- and DMSO-dHL-60 cells on dot-plots; B) Mean FSC and SSC values of studied cells; C) Area of cell cytoplasm and nucleus according to cell staining for F-actin (GFP, green), and nuclei (DAPI, blue); D) Hematoxylin and eosin-stained PMNs, ATRA- and DMSO-dHL-60 cells; E) Representative images of studied cells stained for Arginase (red), F-actin (GFP, green), and nuclei (DAPI, blue); F) Apoptotic and death rates of HL-60, ATRA- and DMSO-dHL-60 cells, determined at day 5 of differentiation; G) Proliferation rate of HL-60, ATRA- and DMSO-dHL-60 cells, presented as percentage of Ki-67-positive cells on day 5 of differentiation. One-way ANOVA with Tukey’s multiple comparisons test was performed to analyzed the differences between experimental groups.

First, we analyzed morphology of the studied cells. To determine cell size and granularity by flow cytometry, the following strategy was applied: initially, doublets were excluded and total number of cells were selected based on FSC (forward scatter) versus SSC (side scatter) distribution (Fig 2A). PMNs were gated based on cell size and CD15 + /CD16^high^ expression. For dHL-60 cell gating, an alternative method based on FSC and SSC was used, as these cells represent a homogeneous cell line without the mixed granulocyte populations (eosinophils and basophils) present in primary PMNs. The dHL-60 cells appeared larger in size compared to PMNs, with the ATRA-dHL-60 cells being the largest (Fig 2B). A similar size difference was observed using microscopy (Fig 2C). More pronounced differences were observed in terms of cell granularity: while PMNs, as expected, exhibited high granularity, the dHL-60 cells lacked granularity (Fig 2B). These cytometric results were further confirmed through microscopy (Fig 2D, E).

Given that cell differentiation and proliferation show a remarkable inverse relationship, we analyzed rates of proliferation and apoptosis in dHL-60 cells. Differentiation of HL-60 cells caused significant increase in cell death. DMSO-dHL-60 cells exhibited the lowest viability, followed by ATRA-dHL-60 cells. Conversely, apoptotic rates were highest in ATRA-dHL-60 cells, followed by DMSO-dHL-60 cells (Fig 2F). Additionally, the ATRA-differentiated cells were the least proliferative, which might indicate their reduced capacity for sustained growth or differentiation under these conditions (Fig 2G). Interestingly, the DMSO-differentiated cells showed no significant difference in proliferation compared to the non-differentiated cells.

### Expression profiling of PMNs and dHL-60 cells

**Fig 3:**
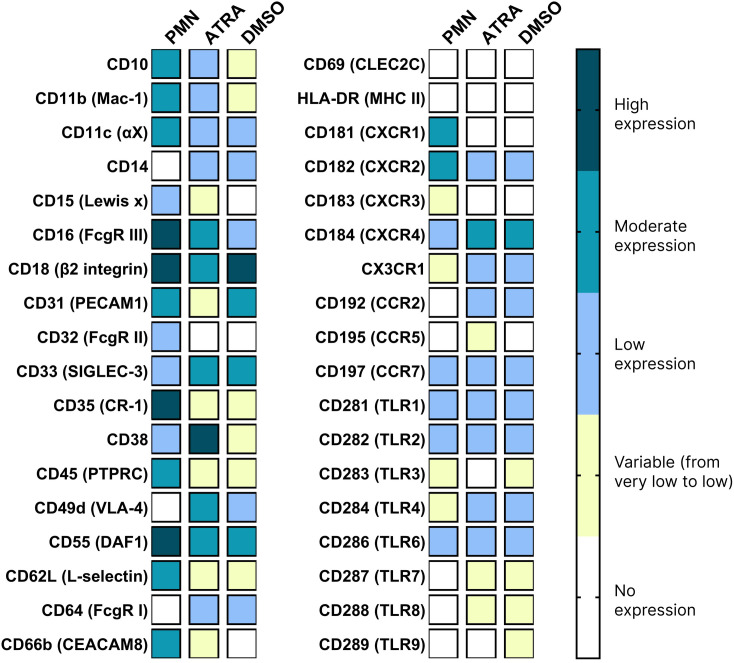
Heat map of surface and intracellular expression levels on primary PMNs and ATRA- or DMSO-differentiated HL-60 cells. Marker expression was assessed by flow cytometry and quantified based on mean fluorescence intensity (MFI).

We conducted a comprehensive screening of surface and intracellular CD markers known to be spontaneously or inducibly expressed by primary PMNs to identify those which are available for phenotypic characteristics of ATRA- and DMSO-dHL-60 cells. A comparative analysis of MFI and percentage of cells positive for the markers was done. Figs 3, S1 and S3 Table in S1 File represent data on expression degree (low, moderate, and high) of surface and intracellular CD markers on the studied cells. As seen from Fig 3, there were different expression patterns among evaluated cell populations. Except for certain TLRs and CCR7, the studied cell populations exhibited distinct expression patterns when compared to one another.

First of all, the results point to the reduced expression of key neutrophil markers in dHL-60 cells. Namely, ATRA- and DMSO-dHL-60 cells exhibited reduced expression of CD11b (Mac-1), CD15, CD16 (FcgRIII) and CD66b. The reduction is more pronounced in DMSO-differentiated cells, which exhibit minimal to no expression of these markers, indicating incomplete or altered differentiation compared to ATRA-ones. CD49d (VLA-4), an adhesive molecule, was absent on PMNs, while dHL-60 cells exhibited higher expression, with the highest levels observed in ATRA-differentiated cells.

Another group of surface markers with altered expression was adhesion molecules, such as CD11c (integrin alpha X), CD31 (PECAM-1), CD62L (L-selectin) and CD18 (β2 integrin), which were expressed across all three cell types, although the levels vary. CD11c and CD62L expression, important for neutrophil rolling and migration, was lower in differentiated cells, which may impact their ability to migrate effectively. Expression of CD31 and CD18 was lower only in the ATRA-dHL-60 cells.

Fc receptors (FcRs) enable neutrophils to recognize and respond to pathogens coated with antibodies. FcRs such as FcγRIII (CD16), FcγRII (CD32), and FcγRI (CD64) were expressed on the surface of the studied cells with varying levels. Expression of CD16 (FcγRIII) is initiated between the metamyelocyte and band stages of neutrophil maturation [15]. Notably, PMNs exhibited high expression of CD16, while ATRA-dHL-60 cells retained moderate levels, and DMSO-dHL-60 cells showed reduced or no expression. This suggests a functional decline in antibody-mediated cytotoxicity by differentiated cells. In contrast to the absence of CD64 (FcγRI) expression on PMNs, its expression was observed on both ATRA- and DMSO-dHL-60 cells. CD32 expression was absent in the dHL-60 cells.

Remarkable differences in surface expression were detected for complement receptor type 1 (CR1) CD35. Its expression in dHL-60 cells was not equally distributed within cell populations. Specifically, 37 ± 8.0% of ATRA- and 58 ± 11.6% of DMSO-dHL-60 cells exhibited positive expression of CD35, whereas PMNs were completely positive for this marker. The low to moderate expression of CD35 on dHL-60 cells suggests a potential impairment in their ability to bind and clear immune complexes and opsonized pathogens.

The migratory abilities of dHL-60 cells are diminished, as evidenced by the low expression of chemokine receptors CXCR1 (CD181) and CXCR2 (CD182). However, CXCR4 (CD184), which is critical for migration to bone marrow, was expressed at high levels on dHL-60 cells, indicating that these cells have the ability to respond to CXCL12 signaling. Furthermore, the elevated CXCR4 expression supports the notion that dHL-60 cells either maintain an immature phenotype or mirror the behavior of aged neutrophils returning to the bone marrow [16].

Expression levels of surface and intracellular TLRs were relatively consistent across all cell types, although ATRA-dHL-60 cells showed slightly higher expression of certain TLRs (e.g., TLR1, TLR4), indicating that these cells may exhibit some innate immune capabilities. Notably, PMNs lacked TLR7 and TLR8 expression, whereas both dHL-60 cell types displayed measurable levels of TLR7 (11.6% ATRA and 17% DMSO) and TLR8 (10% ATRA and 15% DMSO), with ATRA- and DMSO-dHL-60 cells showing comparable expression patterns. Interestingly, cyclic ADP ribose hydrolase (CD38) was the most prominently expressed on ATRA-dHL-60 cells, which is often associated with cellular activation and differentiation, suggesting that ATRA may induce a more activated phenotype compared to DMSO. Markers like HLA-DR (MHC class II) and CD69, typical for activated immune cells, are absent across all cell types, indicating that these cells are not fully activated in terms of antigen presentation.

### Functional activity of primary PMNs and dHL-60 cells

**Fig 4:**
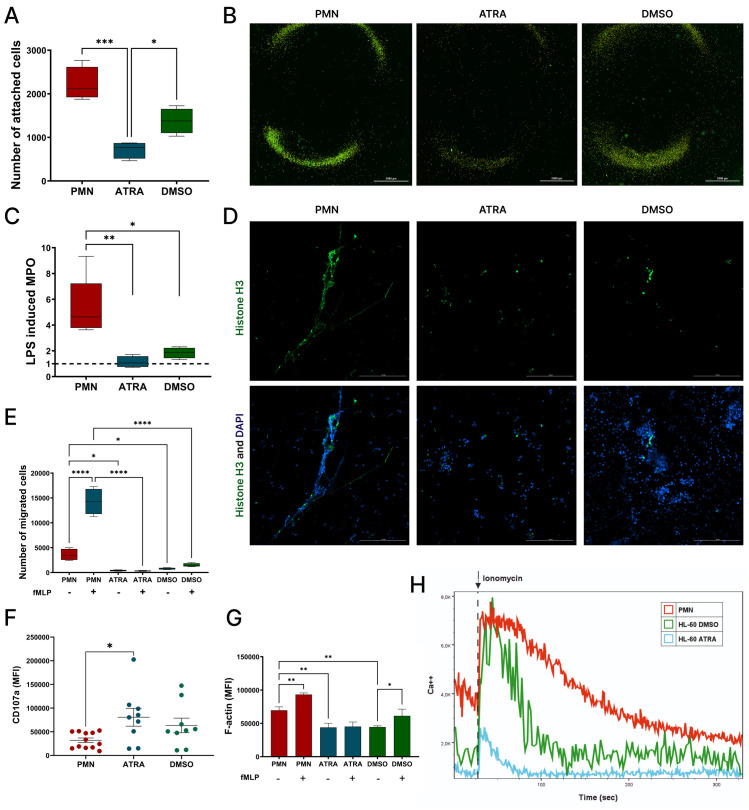
Functional capacities of PMNs and dHL-60 cells. A) Adhesive abilities of studied cells to HeLa cells; B) Adhesive abilities of the cells visualized with fluorescent microscope. Green fluorescence indicates CFSE-labeled PMNs and dHL-60; C) LPS-induced mRNA levels of *MPO* gene. Data was calculated according to the 2^-ΔΔCt^ method and presented as a fold change of LPS-stimulated to unstimulated values for each cell type; D) Representative confocal immunofluorescence microscopy of NETs in PMA-stimulated cells stained for Histone H3 (green) and DAPI (blue); E) Migration rates of the cells passing Transwell inserts spontaneously and induced by fMLP; F) Basal expression of intracellular CD107a measured with flow cytometry; G) Basal and fMLP-induced actin polymerization in the studied cells measured by intracellular Phalloidin staining using flow cytometry; H) Representative histograms of Ca² ⁺ flux dynamics, with the time point of ionomycin addition indicated by an arrow.

Given that dHL-60 cells spontaneously express adhesion molecules such as CD11c, CD11b, CD18, CD31, and CD49d (Fig 3), we aimed to correlate their surface expression with the ability to adhere to a monolayer of HeLa cells. As a result, the highest adhesion was observed for the primary PMNs, which was 10-fold and 3-fold greater than adhesion of ATRA-HL60 and DMSO-HL60 cells, respectively (Fig 4A). Adhesive properties of ATRA-dHL60 cells were minimal within studied cells. In fact, the low ability of ATRA-dHL-60 cells to adhere was correlated with low expression levels of platelet endothelial cell adhesion molecule (PECAM-1) CD31 which contributes to extravasation of the cells to the sites of inflammation [17].

Next, we analyzed the migratory ability of the cells using Transwell inserts. Despite expressing chemokine receptors such as CXCR4, CXCR2, CCR2, and CCR7 dHL-60 cells failed to migrate spontaneously or in response to the stimulus fMLP (Fig 4E). As expected, PMNs exhibited the highest migration rates. The migration rate of DMSO-dHL-60 cells was 2-fold higher than that of ATRA-treated cells, although the difference was not statistically significant. To gain further insight into the observed differences in migratory behavior, we analyzed actin dynamics, as actin polymerization is a critical driver of cell motility. We assessed both spontaneous and fMLP-stimulated actin polymerization in the studied cells. The results revealed that DMSO-dHL-60 cells exhibited significantly stronger actin polymerization responses to fMLP compared to ATRA-dHL-60 cells (~1.4 fold), suggesting that their higher migratory capacity may be attributed to more efficient cytoskeletal remodeling (Fig 4G).

Another antimicrobial strategy employed by PMNs is the formation of NETs, which are released by highly activated cells. We tested formation of NETs by the cells adherent to the glass (Fig 4D), using PMA as a potent inducer. Notably, DMSO was the more efficient agent differentiating HL-60 cells into NET-forming cells compared to ATRA. Despite this, NET formation in DMSO-dHL60 cells was still less efficient when compared with PMNs. To further validate and confirm the NET formation data, we assessed mRNA levels of MPO, a key marker associated with NET formation. As shown in Fig 4C, MPO transcripts were most highly expressed in LPS-stimulated PMNs, showing a 5-fold increase over non-stimulated controls. DMSO-dHL-60 cells exhibited a moderate (~2-fold) increase, whereas ATRA-dHL-60 cells showed no significant induction.

Lysosomal activity was traced by the intracellular expression levels of CD107a, which were measured with flow cytometry. Interestingly, intracellular levels of CD107a markers were significantly lower in primary PMNs in comparison to both ATRA- and DMSO differentiated cells (2.5-fold and 2-fold, correspondingly) (Fig 4F).

Intracellular calcium signaling plays a critical role in cell activation, signaling, and functional responses, therefore, we measured the intracellular levels of Ca^2+^. As expected, the highest levels were expectedly found in PMNs, followed by DMSO-dHL-60 cells (Fig 4H). Interestingly, ATRA-dHL-60 cells exhibited the lowest Ca² ⁺ influx, despite their more mature and activated phenotype. This apparent discrepancy may be explained by several mechanisms. ATRA-dHL-60 cells might prioritize transcriptional and phenotypic maturation over calcium-dependent rapid response mechanisms like chemotaxis, which is more prominent in less mature or DMSO-treated cells. Additionally, ATRA treatment may alter calcium homeostasis by enhancing buffering capacity, reducing store-operated calcium entry, or upregulating calcium extrusion mechanisms, thereby limiting detectable cytosolic Ca² ⁺ increase [18].

### LPS-driven activation and response of PMNs and dHL-60 cells

**Fig 5:**
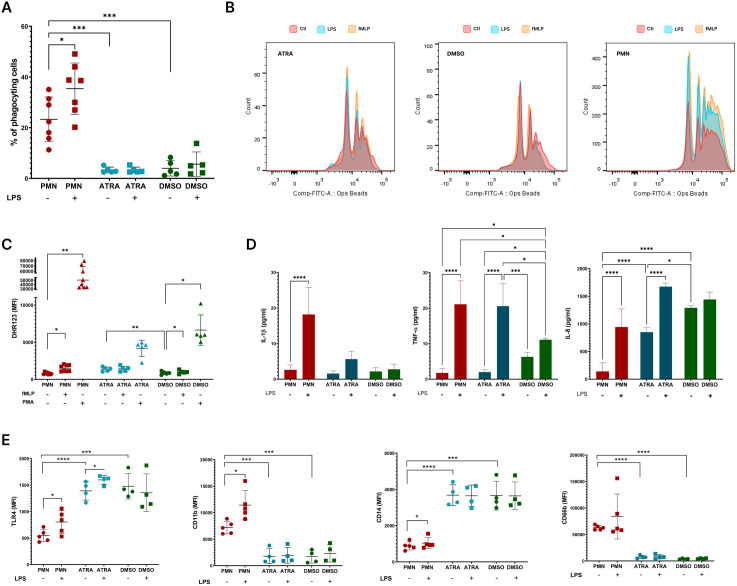
Effect of LPS on dHL-60 cells. A) Phagocytic ability measured by pHrodo particles given to the cells for 1 hour; B) Representative histogram overlays showing phagocytic uptake of fluoresbrite YG microspheres by the studied cells; C) Basal and stimulated ROS burst detected by DHR; D) Cytokine release by studied cells in response to LPS; E) Basal and LPS-stimulated surface expression of CD11b, CD14, CD66b and TLR4 on PMNs, ATRA- and DMSO-dHL-60.

Given that LPS is a key microbial stimulus that activates PMNs through TLR4 signaling, we analyzed LPS-induced activation of the studied cells to assess their ability to generate an antimicrobial response, including phagocytosis and ROS production. The results demonstrated that the spontaneous and induced phagocytic activity of dHL-60 cells was lower than that of PMNs (Fig 5A, B). Additionally, LPS was unable to induce a response in dHL-60 cells. In contrast, the spontaneous oxidative burst activity of both types of dHL-60 cells was comparable to that of PMNs (Fig 5B). While PMA-induced ROS activity was highest in PMNs, dHL-60 cells retained the ability to respond to stimuli. Among the dHL-60 subsets, DMSO dHL-60 cells exhibited slightly stronger ROS activity compared to ATRA-dHL-60 cells. fMLP proved to be a weak inducer of this response (Fig 5C).

We also assessed the ability of the cells stimulated with LPS to produce soluble mediators. Both ATRA- and DMSO-differentiated HL-60 cells produced comparable spontaneous levels of IL-1β and TNF-α, matching those observed in PMNs. The only exception was TNF-α spontaneous levels which were higher in DMSO-differentiated cells (Fig 5D). Notably, spontaneous IL-8 levels were even higher in the supernatants of both ATRA- and DMSO-dHL-60 cells compared to those from PMNs. Following LPS stimulation, PMNs showed the strongest response with the highest IL-1β production, while dHL-60 cells failed to upregulate IL-1β in response to the pro-inflammatory stimulus. Among the HL-60 cells, ATRA-dHL-60 cells exhibited a more pronounced response, comparable to that of primary PMN, with an increased production of TNF-α, and IL-8, supporting the idea that ATRA-dHL-60 cells achieve a more activated state than DMSO-dHL-60 cells.

Phenotypically, LPS caused an up-regulation of surface markers CD11b, CD14, and TLR4 in PMNs. Surprisingly, despite the ability of dHL-60 cells to produce cytokines in response to LPS, the phenotype of the cells remained unchanged. The only exception was TLR4, which showed up-regulation on ATRA-dHL-60 cells in response to LPS (Fig 5E).

### Influence of studied cells on proliferation of tumor growth

**Fig 6:**
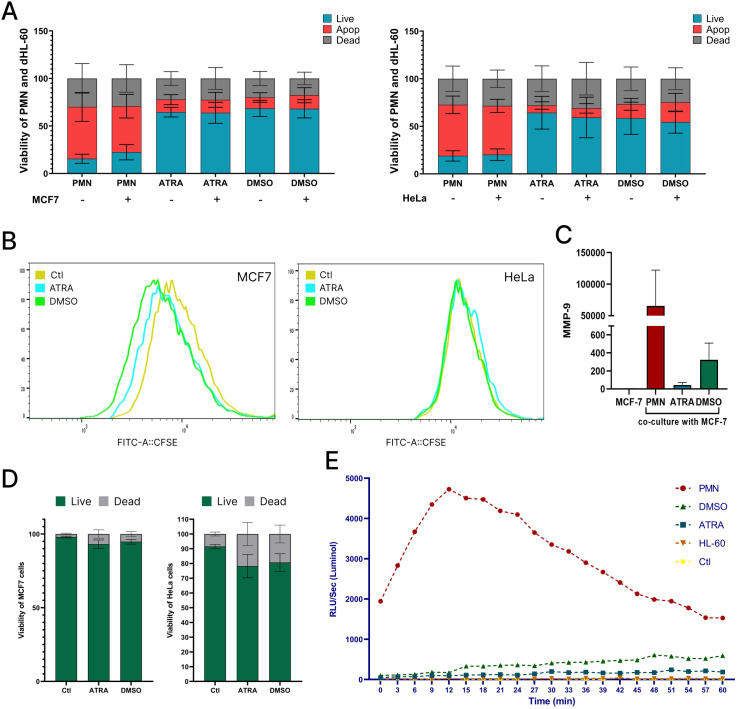
Interplay between tumor cells and dHL-60 cells. A) Percentage of viable, apoptotic and dead PMNs, ATRA- and DMSO-dHL-60 after overnight co-cultivation with MCF-7 or HeLa cells; B) Representative histograms of MCF-7 and HeLa cell proliferation (measured with CFSE) after 3-day co-cultivation with ATRA- and DMSO-dHL-60; C) Predictive concentrations of MMP-9 measured in supernatants after 2-day co-culture of PMNs, ATRA- and DMSO-dHL-60 cells with MCF-7 cells. MCF-7 cells cultured alone served as a control. Measurements were performed using a custom multiplex assay; D) Percentage of viable and dead MCF-7 and HeLa cells co-cultured with ATRA- and DMSO-dHL-60 cells for 3 days; E) Kinetics of ROS production in PMA-stimulated cells cultured in the presence of MCF-7-conditioned medium, measured using luminol-enhanced chemiluminescence. Reactive oxygen species (ROS) generation was monitored at 2-minute intervals and expressed as relative light units (RLU). The conditioned medium was collected from MCF-7 cells. Unstimulated cells served as negative controls. The start point indicates the time of PMA addition. Data were normalized to ROS levels in unstimulated cells.

To investigate the impact of dHL-60 cells on tumor growth, we assessed their ability to modulate tumor cell (MCF-7 and HeLa) proliferation in a co-culture system. Specifically, we evaluated their effects on tumor cell viability, apoptosis, and proliferation. By comparing dHL-60 cells and PMNs, we aimed to determine whether differentiation status influences their functional interactions with tumor cells. First, we analyzed the survival rates of dHL-60 cells after overnight co-culture with MCF-7 and HeLa cells. The viability patterns of dHL-60 cells remained similar across conditions, with comparable apoptotic rates and a consistent percentage of live cells (Fig 6A). As expected, the majority of PMNs did not survive overnight co-culture, with only 15−19% of PMNs remaining viable. Interestingly, PMNs exhibited improved survival when co-cultured with MCF-7 cells, reaching 22,5 ± 3,6%, compared to 15,5 ± 2% when cultured alone (Fig 6A). This observation reflects the higher maturation stage of PMNs and their enhanced capacity to interact with and potentially respond to tumor cells.

Long-term co-cultivation with tumor cells was conducted exclusively with dHL-60 cells. When assessing the viability of tumor cells co-cultured with dHL-60 cells (overnight, 2 and 3 day cultivation), MCF-7 cells exhibited higher survival rates compared to HeLa cells. Notably, the number of live HeLa cells decreased by 13 and 11% when co-cultured with ATRA- and DMSO-dHL-60 cells, correspondingly (Fig 6D).

Neither ATRA- nor DMSO-dHL-60 cells significantly affected HeLa proliferation, while MCF-7 cells exhibited altered proliferation rates in their presence. Notably, co-culture with DMSO-dHL-60 cells provided MCF-7 cells with stronger growth signals, as evidenced by an increased proliferation rate after 3 days, which were higher than ATRA ones (Fig 6B). To investigate potential mechanisms underlying the stronger growth-promoting effects of DMSO-dHL-60 cells, we assessed extracellular ROS production and MMP9 release in co-cultures of the studied in the presence of MCF-7 cells. DMSO-dHL-60 cells exhibited markedly higher ROS activity compared to ATRA-dHL-60 cells (Fig 6C, E). Similarly, MMP-9 release was higher in DMSO-dHL-60 cells compared to ATRA-dHL-60 cells. TGF-β levels were below the detection threshold of the assay.

All experimental findings comparing ATRA- and DMSO-differentiated HL-60 cells are summarized in S4 Table in S1 File, which provides an overview of their phenotypic, transcriptional, and functional characteristics.

## Discussion

The appropriate use of cell lines as models for primary cells is important in different biological experiments. As such, the degree to which the phenotype expressed by cell lines represents that of the primary system is of central relevance. In this study, we aimed to thoroughly evaluate the differentiation potential of ATRA and DMSO, as widely accepted differentiation factors for HL-60 cells. Our findings provide insight into both the similarities and differences in how these agents affect HL-60 cell activation, as well as the implications for their functional abilities.

Our phenotypic and morphological analysis confirmed that both ATRA- and DMSO-dHL-60 cells exhibit immature promyelocyte stage of neutrophil development [19]. Specifically, their morphology, along with the increased expression of well-established maturation markers CD49d and low expression of CD10, CD11b, CD11c, CD16, CD35, and CD66b, indicate that both dHL-60 cell populations phenotypically resemble immature neutrophils. Differentiation with ATRA resulted in a higher basal expression of C/EBPα compared to DMSO-treated cells. As key regulators of early myeloid development, C/EBPα and PU.1 play crucial roles in lineage commitment. Elevated C/EBPα expression in myeloid progenitors lacking PU.1 drives granulocytopoiesis, whereas PU.1 induction by C/EBPα during hematopoiesis promotes monocyte differentiation [20–22]. Consistent with our findings, lack of PU.1 expression alongside higher C/EBPα levels suggests a commitment toward granulopoiesis. However, the sustained elevation of C/EBPα mRNA levels in ATRA-differentiated cells suggests a more advanced stage of maturation. Similarly, ATRA differentiation results in a more activated and mature phenotype compared to DMSO-dHL-60 cells. Reduced proliferation rate of ATRA-dHL-60 cells suggests a more advanced differentiation process associated with increased apoptosis and higher expression of CD11b, CD16 and CD38, whereas DMSO-dHL-60 cells exhibited a lesser degree of these changes [23,24].

Functionally, both studied cell types exhibited low to moderate immune responses. ATRA-differentiated cells showed minimal phagocytic activity and ROS production, even in response to LPS, although they exhibit elevated production of TNF-α and IL-8, likely mediated through TLR4-signalling. Our findings contrast with a previous study by Manda-Handzlik et al, which reported that ATRA-dHL-60 cells efficiently phagocytosed *E. coli* at levels comparable to PMNs [13]. The difference may lie in the distinct mechanisms of particle uptake, including macropinocytosis or mannose receptor-mediated pathways, as well as differences in the degree of neutrophilic maturation achieved during differentiation. Low phagocytic abilities are supported by the low ROS activity, absence of specific granules, and lack of responsiveness to LPS. These observations suggest that while ATRA-dHL-60 cells are transcriptionally primed for inflammatory cytokine production, their phagocytic and oxidative capabilities remain limited. Despite exhibiting a more advanced transcriptional and phenotypic profile indicative of maturation, ATRA-differentiated HL-60 cells appear to be functionally less mature, showing limited capacity for key effector responses such as phagocytosis and ROS production.

DMSO-differentiated cells, while exhibiting a less activated transcriptional profile, displayed overall moderate functional responses. This apparent discrepancy may be attributed to differences in the maturation trajectory induced by each agent, where ATRA drives terminal differentiation focused on gene expression and surface marker acquisition, DMSO may favor the development of functional effector mechanisms without fully engaging transcriptional programs associated with neutrophil activation. Their inability to produce cytokines in response to LPS, along with lower surface expression of Fc and complement receptors, suggests a more immature or quiescent state. Despite this, DMSO-dHL-60 cells uniquely demonstrated the ability to form NETs upon stimulation, albeit less efficiently than primary PMNs. Supporting this, DMSO-dHL-60 cells exhibited increased mRNA levels of MPO in response to LPS and a greater ability to produce ROS in response to PMA, both key contributors to NET formation. [2,25,26]. This indicates that DMSO-differentiated cells retain some neutrophil-like features, particularly those involved in antimicrobial defense, despite being less responsive to inflammatory stimuli compared to ATRA-differentiated cells. Notably, DMSO-dHL-60 cells exhibit a higher rate of cell death compared to ATRA-differentiated cells. The elevated levels of NET formation observed in these dying cells may suggest that a significant proportion of the cell death may occur via NETosis. Another important functional feature is the difference in the migratory and adhesive abilities of the studied cells. Both ATRA- and DMSO-dHL-60 cells exhibited significantly lower adhesion to epithelial monolayers and reduced migration in response to chemotactic stimuli compared to PMNs. The low expression of key adhesion molecules such as CD62L and CD31 in dHL-60 cells likely impairs their ability to extravasate and migrate effectively. However, DMSO-dHL-60 cells exhibited stronger abilities to adhere and migrate in comparison to the ATRA-differentiated ones, likely due to their higher expression of CD18 and CD31. Moreover, the tumor-promoting potential of DMSO-dHL-60 cells shown in current study may be driven by elevated production of angiogenic MMP-9 and ROS. Neutrophil-derived MMP-9 has been shown to contribute to cancirogenesis by degrading extracellular matrix components, facilitating tumor invasion, and accelerating angiogenesis through the release of matrix-bound pro-angiogenic factors such as vascular endothelial growth factor (VEGF) [27]. In parallel, excessive ROS production can promote genomic instability, support tumor cell proliferation, and modulate the tumor microenvironment in favor of cancer progression. These combined effects may underlie the pro-tumorigenic behavior observed in the presence of DMSO-dHL-60 cells. ROS mediate the release of NETs via an NADPH oxidase 2-independent pathway, potentially involving mitochondrial ROS or alternative sources such as myeloperoxidase and calcium-dependent signaling, which can trigger chromatin decondensation and NETosis under certain pathological conditions. Enhanced NET formation, potentially driven by excessive ROS, and the subsequent release of NETosis-derived factors have been linked to tumor progression and metastasis by promoting a pro-thrombotic environment, aiding tumor cell evasion from immune responses, and facilitating their adhesion and colonization at distant sites [2,28].

Interestingly, both dHL-60 cells exhibited CD62L^low^/CXCR4^high^ phenotype which is characteristic of aged or reverse-migrated neutrophils [29,30]. Studies have also shown that CXCR4 upregulation and CD62L shedding are associated with an overly activated neutrophil phenotype, often linked to enhanced NET formation [31,32]. Differentiation could induce stress-related pathways that mimic neutrophil aging or immunosuppression states leading to increase of CXCR4 expression [33,34]. The phenotype observed in our differentiated cells partly bears similarities to that of myeloid-derived suppressor cells (MDSCs), which exhibit a CD62L^low^/CXCR4^high^ profile and promote immune evasion by suppressing effector immune cells [35]. Thus, both differentiation models are suitable for tumor investigation, especially considering their distinct phenotypic and functional characteristics.

In summary, different differentiation conditions significantly influence the functional specialization of HL-60 cells. ATRA-differentiated cells appear to follow a more conventional neutrophil-like maturation path with moderate activation, while DMSO-differentiated cells retain a more immature phenotype yet demonstrate unique functional properties such as enhanced NET formation. The distinct properties of ATRA- and DMSO-dHL-60 cells could be relevant for disease modeling. ATRA-dHL-60 cells may better represent functionally restrained neutrophils in chronic inflammation, whereas DMSO-dHL-60 cells might be more applicable for studying NETosis-driven pathologies, such as autoimmune diseases, thrombotic disorders and cancer. These insights provide valuable guidance for selecting appropriate differentiation conditions in experimental and translational research.

## Supporting information

S1 File S1 Table. List of conjugated antibodies used in the study. S2 Table. Primer sequences. S1 Fig. Representative histogram overlays of CD markers as analyzed by flow cytometry. S3 Table. Basal surface and intracellular expression levels of key neutrophilic markers on PMNs and ATRA- or DMSO-differentiated HL-60 cells. S4 Table. A summary of all phenotypic and functional differences between ATRA- and DMSO-differentiated HL-60 cells.
(DOCX)
