# Supplementary material for: Unraveling the functional landscape of ATRA- and DMSO-differentiated HL-60 cells
Source: PLoS One. 2025 Sep 8;20(9):e0331783. doi: 10.1371/journal.pone.0331783 (PMC12416653; doi:10.1371/journal.pone.0331783)
Supplement: S1 File — S1 Table. List of conjugated antibodies used in the study. S2 Table. Primer sequences. S1 Fig. Representative histogram overlays of CD markers as analyzed by flow cytometry. S3 Table. Basal surface and intracellular expression levels of key neutrophilic markers on PMNs and ATRA- or DMSO-differentiated HL-60 cells. S4 Table. A summary of all phenotypic and functional differences between ATRA- and DMSO-differentiated HL-60 cells. (DOCX) [file pone.0331783.s001.docx]

**Unraveling the functional landscape of ATRA- and DMSO-differentiated HL-60 cells**

Susanna Ghonyan^1^, David Poghosyan^1^, Anush Martirosyan^1^, Sona Margaryan^1^, Aida Avetisyan^2^, Zaruhi Khachatryan^3^, Gayane Manukyan^1^*****

^1^Laboratory of Molecular and Cellular Immunology, Institute of Molecular Biology, National Academy of Sciences, Yerevan, Armenia;

^2^Laboratory of Cell Biology and Virology, Institute of Molecular Biology, National Academy of Sciences, Yerevan, Armenia

^3^Laboratory of Evolutionary Genomics, Institute of Molecular Biology, National Academy of Sciences, Yerevan, Armenia

**Supplemental Table S1. List of conjugated antibodies used in the study.**

| **Antigen** | **Fluorochrome** | **Producer** |
| --- | --- | --- |
| CD10 | APC | BioLegend |
| CD11b | PerCP-Cy5.5 | BioLegend |
| CD11c | PE-Cy7 | BioLegend |
| CD14 | FITC | BD Biosciences |
| CD15 | PerCP-Cy5.5 | BioLegend |
| CD16 | BV650 | BioLegend |
| CD18 | PE | BioLegend |
| CD31 | APC-Cy7 | BioLegend |
| CD32 | APC | BioLegend |
| CD33 | FITC | BioLegend |
| CD35 | PE | BioLegend |
| CD38 | PE-Cy7 | eBioscience |
| CD45 | PerCP-Cy5.5 | BioLegend |
| CD49d | PE-Cy7 | BioLegend |
| CD55 | PE | BioLegend |
| CD62L | FITC | BioLegend |
| CD64 | APC-Cy7 | BioLegend |
| CD66b | APC | BioLegend |
| CD69 | APC | BioLegend |
| CD107a | APC | BioLegend |
| CD181 (CXCR1) | FITC | BioLegend |
| CD182 (CXCR2) | PE-Cy7 | BioLegend |
| CD183 (CXCR3) | PerCP-Cy5.5 | BioLegend |
| CD184 (CXCR4) | PE | BD Biosciences |
| CD192 (CCR2) | FITC | BioLegend |
| CD195 (CCR5) | APC | BioLegend |
| CD197 (CCR7) | PE-Cy7 | BD Biosciences |
| CX3CR1 | BV785 | BioLegend |
| CD281 (TLR1) | BV421 | BD Biosciences |
| CD282 (TLR2) | PE-Cy7 | BioLegend |
| CD283 (TLR3) | APC | BioLegend |
| CD284 (TLR4) | PE | BioLegend |
| CD286 (TLR6) | PE | BioLegend |
| CD287 (TLR7) | BV421 | BioLegend |
| CD288 (TLR8) | FITC | BioLegend |
| CD289 (TLR9) | APC | BioLegend |
| HLA-DR | BV605 | BD Biosciences |

**Supplemental Table S2. Primer sequences.**

| ***GAPDH*** | *forward* | TCCTGTTCGACAGTCAGCCGCA |
| --- | --- | --- |
|  | *reverse* | GCGCCCAATACGACCAAATCCGT |
| ***PU.1*** | *forward* | TGGAAGGGTTTCCCCTCGTC |
|  | *reverse* | TGCTGTCCTTCATGTCGCCC |
| ***C/EBP-α*** | *forward* | GAATCTCCTAGTCCTGGCTC |
|  | *reverse* | GATGAGAACAGCAACGAGTAC |
| ***NFkB*** | *forward* | CTGTCCTTTCTCATCCCATCTT |
|  | *reverse* | ACACCTCAATGTCCTCTTTCTG |
| ***MPO*** | *forward* | TGGTGGGAGAACGAGGGTGTG |
|  | *reverse* | CGGTGGTGATGCCTGTGTTGTC |

***Supplemental Figure S1.* Representative histogram overlays of CD markers as analyzed by flow cytometry.**


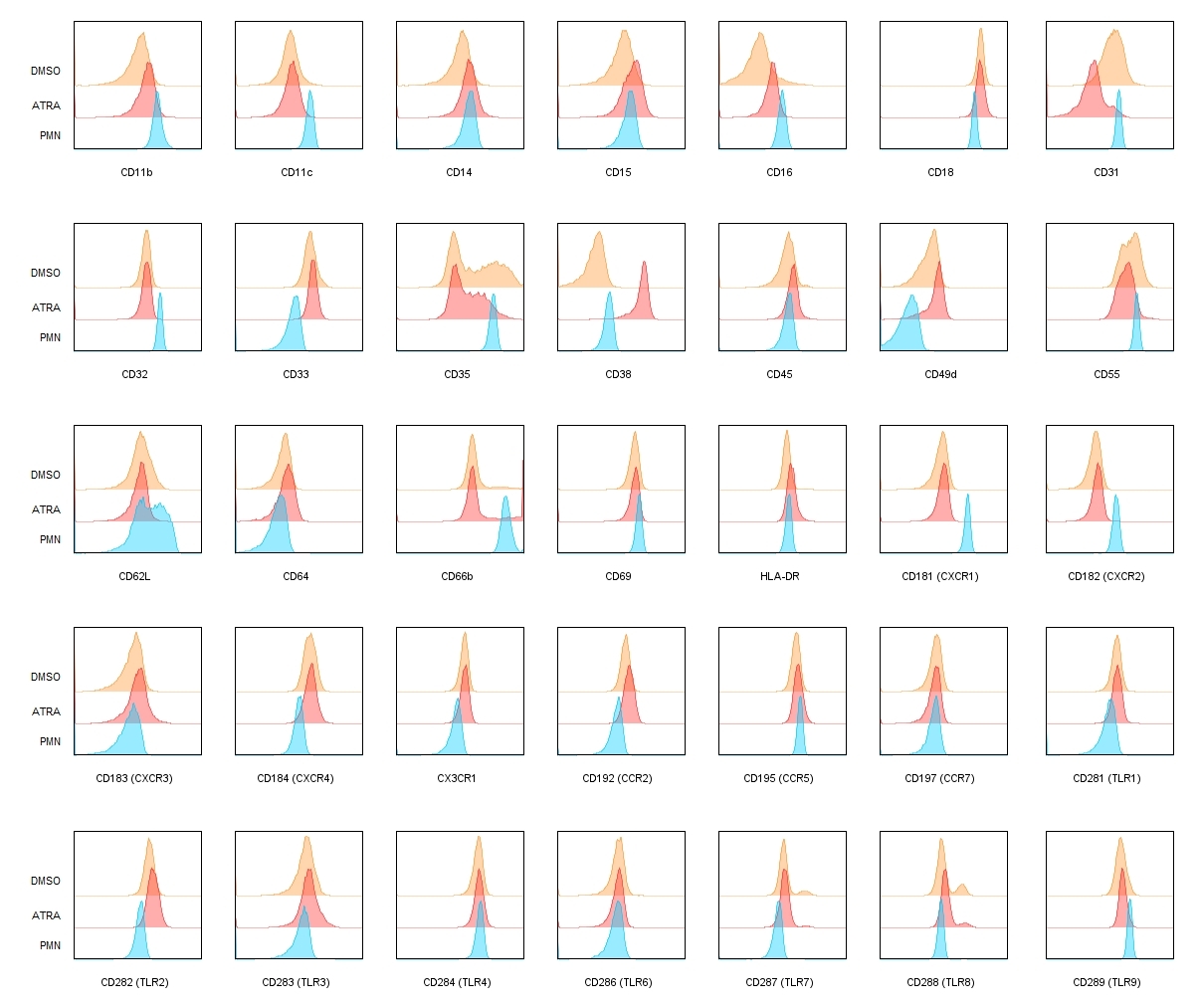


**Supplemental Table S3. Basal surface and intracellular expression levels of key neutrophilic markers on PMNs and ATRA- or DMSO-differentiated HL-60 cells.** Expression levels are presented as (-) for absence of expression and (+) positive expression, identified based on mean fluorescence intensity of marker expression. Mixed-effects model analysis was performed to analyze differences between experimental groups. The Tukey post-hoc test was performed to compare every mean with every other mean. Data represent the average of [n=4] independent experiments.

* – differences between dHL-60 cells and PMNs. * p<0.05; ** p<0.01; *** p<0.001
# – differences between ATRA- and DMSO-dHL-60 cells. # p<0.05; ## p<0.01; ### p<0.001

a – data calculated and presented as the percentage of positive cells.

| **Surface marker** | **Alternat.**  **name** | **PMN** | **ATRA** | **DMSO** |
| --- | --- | --- | --- | --- |
| CD10 |  | ++ | + | -/+ |
| CD11b | Mac-1, CR-3, αM | ++ | +* | -/+** |
| CD11c | αX | ++ | +** | +** |
| CD14 |  | - | ++* | + ^#^ |
| CD15 | Lewis x | + | -/+ | - ^#^ |
| CD16 | FcgR III | +++ | ++* | +*** ^#^ |
| CD18 | LFA-1, β2 integrin | +++ | ++ | +++ |
| CD31 | PECAM1 | ++ | -/+ | ++ ^#^ |
| CD32 | FcgR II | + | **-***** | **-**** |
| CD33 | SIGLEC-3 | + | ++* | ++* |
| CD35 | CR-1 | +++  (99.3±0.3)^a^ | -/+*  (36.7±8.0)^a^ ** | -/+  (58.4±11.6)^a^ * |
| CD38 |  | + | +++** | -/+ ^#^ |
| CD45 | PTPRC | ++ | -/+* | -/+ ^#^ |
| CD49d | VLA-4 | - | ++ | + |
| CD55 | DAF1 | +++ | ++* | ++ |
| CD62L | L-selectin | ++ | -/+ | -/+ |
| CD64 | FcgR I | - | + | + |
| CD66b | CEACAM8 | ++  (99.1±0.32)^a^ | -/+*  (22.77±4.7)^a^ *** | -* ^#^  (9.58±2.8)^a^ *** |
| CD69 | CLEC2C | - | - | - |
| HLA-DR | MHC II | - | - | - |
| CD181 | CXCR1 | ++ | -* | -* |
| CD182 | CXCR2 | ++ | +* | +* |
| CD183 | CXCR3 | -/+ | -*** | -** |
| CD184 | CXCR4 | + | ++** | ++** |
| CX3CR1 |  | -/+ | +* | +** |
| CD192 | CCR2 | - | +*** | -/+*** ^###^ |
| CD195 | CCR5 | - | -/+ | - ^##^ |
| CD197 | CCR7 | + | + | + |
| CD281 | TLR1 | + | +** | +** |
| CD282 | TLR2 | + | + | + ^#^ |
| CD283 | TLR3 | -/+ | - | -/+ ^#^ |
| CD284 | TLR4 | -/+ | + | + |
| CD286 | TLR6 | + | + | + |
| CD287 | TLR7 | -/+  (2.8±0.4)^a^ | -/+*  (11.7±2.5)^a^ * | -/+*  (16.7±4.2)^a^ * |
| CD288 | TLR8 | -/+  (2.5±0.4)^a^ | -/+*  (10.0±2.7)^a^ | -/+  (15.3±3.7)^a^ * |
| CD289 | TLR9 | - | - | -/+*** |

**Supplemental Table S4.** A summary of all phenotypic and functional differences between ATRA- and DMSO-differentiated HL-60 cells.

|  | **ATRA** | **DMSO** |
| --- | --- | --- |
|  | ***Maturation markers*** | |
| Maturation-associated gene expression | **Higher** |  |
| Intracellular protein levels of Arginase 1 | **Higher** |  |
| Expression levels of maturation surface markers (phenotype) | **Higher** |  |
| Cell proliferation |  | **Higher** |
|  | ***Functional maturation*** | |
| Inducible ROS |  | **Higher** |
| Phagocytosis in response to activating signals | **Comparable** | **Comparable** |
| Chemotaxis |  | **Higher** |
| Cytoskeleton mobilization |  | **Higher** |
| Netosis |  | **Higher** |
| Ca2+ mobilization |  | **Higher** |
| Pro-inflammatory cytokine production | **Comparable** | **Comparable** |
| Tumor-promoting activity |  | **Higher** |
